# Case Report: Clinical Analysis of Seven Neonates With Prader-Willi Syndrome and Review of the Literature

**DOI:** 10.3389/fped.2021.633532

**Published:** 2021-02-18

**Authors:** Yu Hu, XinDong Xue, JianHua Fu

**Affiliations:** Department of Pediatrics, Shengjing Hospital of China Medical University, Shenyang, China

**Keywords:** clinical features, genetic testing, infant, neonate, Prader-Willi syndrome

## Abstract

**Objective:** The clinical symptoms of neonatal Prader-Willi syndrome (PWS) are not typical and are easy to miss. The aim of the study was to investigate the clinical features and genetic characteristics of seven cases of neonatal PWS from northern China, and to improve the understanding of PWS in neonates.

**Methods:** We retrospectively analyzed seven infants diagnosed by methylation specific multiplex ligation probe amplification technology (MS-MLPA) in the Neonatology Unit of Shengjing Hospital of China Medical University from September 2016 to July 2020.

**Results:** All seven cases involved full term or nearly full-term infants born to mothers without a history of abnormal pregnancy or delivery. Difficulty in feeding occurred immediately after birth in infants with decreased hypotonia. Five patients had characteristic craniofacial morphology, such as a prominent forehead, narrow face, almond-shaped eyes, small mouth, and downturned mouth. Further, three of the seven infants had patent ductus arteriosus (PDA). In addition, three neonates had hyperammonemia, hypoglycemia, and idiopathic edema, respectively. PWS could be effectively diagnosed and genotyped by MS-MLPA.

**Conclusion:** Neonates with PWS have hypotonia and feeding difficulty. Characteristic facial features and genital hypoplasia are common in neonatal PWS. Infants with PWS may be predisposed to PDA, hypoglycemia, hyperammonemia, and edema.

## Introduction

Prader-Willi syndrome (PWS) is a complex neuroendocrine hereditary syndrome first described by John Langdon Down in 1887 and named by Swiss doctors Andrea Prader, Heinrich Willi, and Alexis Labhartin in 1956, based on the clinical characteristics of nine pediatric cases (1). The incidence of PWS varies in the range of 1/10,000–1/30,000 across ethnicities (2). The epidemiological data of PWS is lacking in China. The National Health Commission, the Ministry of Science and Technology, the Ministry of Industry and Information Technology, the State Drug Administration, and the State Administration of Traditional Chinese Medicine jointly issued a list of rare diseases in China. The list included 121 diseases, 18 of which have a neonatal onset. PWS is one of the 18 rare neonatal diseases in the list. The causes and genetic mechanisms in PWS are complex (3). Most cases are due to new mutations, meaning both parents are normal, and only a small proportion of PWS cases are inherited. It occurs due to a genetic defect during the stage of egg (sperm) or embryo formation, which results in the deletion or disruption of more than 10 genes on the long arm of chromosome 15. If the defect is inherited from the father, or if the patient has two maternal copies of chromosome 15 with the defect, the result is PWS.

Prader-Willi syndrome results from the paternally inherited loss of the chromosome region 15q11.2-13 (3), which causes complex influences in appetite, development, metabolism, cognition, and behavior (4). PWS is also the most common cause of genetic obesity (5). Additionally, difficulty with public transport and hyperlexia are two of the lesser known manifestations of PWS (6). There is a continuum in the clinical presentation and physical characteristics of PWS, and variations exist among different age groups (7). In the neonatal stage, marked hypotonia and feeding difficulties are characteristic clinical features. In the infantile stage, patients gradually show developmental delay. In the childhood stage, hyperphagia for high carbohydrate food with an evident satiety is typically observed. Later, in the adolescence stage, the patient usually shows weight gain and several behavioral problems. In fact, most patients seek medical advice only after they have developed obesity (7).

It is important to recognize the features of PWS as soon as possible because of its long-term implications. Since PWS is a complex genetic disorder caused by the lack of expression of paternally active genes on chromosome region 15q11.2-q13, there are three different genetic subtypes of PWS (8): deletion of the paternal copy of 15q11-q13 (70–75% of cases), maternal uniparental disomy (UPD) for chromosome 15 (25–30%), and imprinting defect because of the silencing of paternal alleles (1–3%). Methylation specific multiplex ligation probe amplification (MS-MLPA) is an effective molecular detection method for PWS based on differential methylation status. In this study, we report seven neonatal patients from northern China diagnosed with PWS by MS-MLPA technology and analyzed their clinical features and genetic characteristics. The aim of this study was to strengthen the understanding of PWS in neonates.

## Materials and Methods

### Subjects

We retrospectively analyzed the medical records of seven neonates with PWS (three boys and four girls) who were genetically diagnosed using MS-MLPA. All of them were hospitalized in the Department of Neonatology, Shengjing Hospital of China Medical University from September 2016 to July 2020. The study protocols were approved by the Hospital Ethics Committee for Human Research of Shengjing Hospital of China Medical University.

### Methods

All the seven neonates were diagnosed by MS-MLPA after blood sampling. Abnormal methylation pattern, chromosome microdeletions, and maternal uniparental disomy were analyzed to aid the genetic diagnosis.

### Statistical Analysis

Clinical data on sex, birth weight, gestational age (GA), age of onset, age at hospital admission, first symptoms, clinical magnifications, genotype, age at genetic diagnosis, treatment, and prognosis were collected. All clinical data were descriptively analyzed. Birth weights were presented as means ± standard deviations.

## Results

### General Data

In total, seven neonates (three boys and four girls) were included in the study. The GA ranged from 36 weeks and 6 days to 40 weeks. Birth weight varied from 2.4 to 3.4 kg with an average weight of 2.79 ± 0.35 kg. Maternal ages ranged from 23 to 37 years old. All cases were appropriate for GA. Interestingly none of the seven neonates was a firstborn and all had a maternal history of multiple pregnancies. Breech presentation was observed in one patient. Of note, 71.4% (5/7) were delivered by cesarean section; one of them was delivered in breech position, and another suffered from intrauterine distress and other symptoms. Four patients were second-born children, three of whom had healthy families, and one had a brother suffering from ventricular septal defect. They were all full term or nearly full-term infants without maternal history of abnormal pregnancy or asphyxia at birth. Age at hospital admission varied from 2 days to 16 days. The results are summarized in Table 1.

**Table 1:** Demographical characteristics of the neonates with Prader-Willi syndrome (*n* = 7).

	**Sex**	**Birth weight (g)**	**History of birth**	**Delivery mode**	**Gestational age (weeks)**	**Maternal ages (years)**	**Breech presentation**	**Age of onset**
Case 1	Female	2,500	G3P1	Cesarean	38^+3^	30	–	1 d
Case 2	Male	3,100	G4P2	Cesarean	39^+5^	37	+	3 d
Case 3	Female	2,700	G2P2	Eutocia	40	30	–	1 d
Case 4	Male	3,400	G4P1	Cesarean	38	23	–	1 d
Case 5	Female	2,750	G2P2	Eutocia	37	25	–	1 d
Case 6	Male	2,400	G2P2	Cesarean	36^+6^	33	–	1 d
Case 7	Female	2,700	G2P1	Cesarean	39^+2^	26	–	1 d

### Clinical Features

All the seven cases were Han Chinese. As shown in Table 2, five of them had decreased fetal movement during pregnancy. The first symptom in all cases was a difficulty in feeding immediately after birth. On admission, the physical examination showed a weak crying, poor rooting, sucking and swallowing reflexes in all cases. All the seven neonates had central hypotonia and poor feeding ability. Five cases (71%) had characteristic craniofacial morphology including prominent forehead, narrow face, almond-shaped eyes, small mouth, and downturned mouth. Further, two of the three boys (67%) had bilateral cryptorchidism. The genitalia of one girl (25%) had the appearance of labia minora. Three cases (43%) were diagnosed with patent ductus arteriosus (PDA) by cardiac ultrasonography with a diameter range from 1.5 to 3.3 mm. In addition, three neonates had hyperammonemia, hypoglycemia, and idiopathic edema, respectively. All of them completed the head magnetic resonance and electromyography examination without abnormalities. Another girl unfortunately suffered from nemaline myopathy caused by a mutation in the *NEB* gene.

**Table 2:** Clinical characteristics of the neonates with Prader-Willi syndrome (*n* = 7).

	**Age at hospital admission**	**Decreased fetal movement**	**Hypotonia and feeding difficulties**	**Specific facial features**	**Genital hypoplasia**	**PDA**	**Others**	**Genetic type**	**Age at genetic diagnosis**	**Age at GH treatment start**
Case 1	17 d	+	+	+	+	–	–	Paternal deletion	30 d	6 m
Case 2	5 d	+	+	+	–	–	–	UPD	16 d	6 m
Case 3	2 d	–	+	–	–	–	Nemaline myopathy	Paternal deletion	20 d	–
Case 4	2 d	+	+	–	+	+	Hyperammonemia	UPD	12 m	12 m
Case 5	2 d	–	+	+	–	–	Eyelid and limb edema	Paternal deletion	11 d	6 m
Case 6	6 d	+	+	+	+	+	Hypoglycemia	Imprinting defect	24 d	–
Case 7	2 d	+	+	+	+	+	46XX(16qh+)	Imprinting defect	11 d	6 m

*PDA, patent ductus arteriosus; UPD, uniparental disomy*.

### Genetics

Using the MS-MLPA method, a deletion was detected in three patients (43%). Two patients had a maternal uniparental disomy (29%), and an abnormal methylation pattern without deletion was observed in other two patients (29%). Further, one girl exhibited chromosomal polymorphism, 46XX(16qh+). The results are summarized in Table 2.

### Diagnosis, Treatment, and Outcome

As shown in Table 2, five neonates with PWS were diagnosed between postnatal days 11 and 30, and were discharged when their feeding problems started to improve at 12 to 47 days (average 22 days) of age. Four of them initiated growth hormone (GH) treatment from the age of 6 months. A girl of the five neonates gave up the treatment because of comorbid nemaline myopathy disease. Apart from the five neonates, one case was discharged before laboratory examination and received a genetic diagnosis and GH treatment in a follow-up visit at 1 year old. Another patient was discharged just after genetic diagnosis at postnatal days 24 and died shortly after leaving the hospital because of poor sucking.

## Discussion

Recently, an MS-MLPA kit equipped with suitable discharge was introduced. It can detect gene duplication or deletion, distinguish types of deletion, and estimate the methylation status. MS-MLPA allows to detect large, abnormal, or subtle deletions of chromosomes, but not balanced translocations. MS-MLPA cannot detect any changes that lie outside the target critical region on chromosome 15. The positive detection rate can be as high as 99% (9). In this article, the genetic analysis, which was performed using the MS-MLPA method, showed a deletion of the paternal chromosome 15 in three patients (43%), maternal uniparental disomy in two patients (29%), and imprinting defect in two other patients (29%). Furthermore, their parents and relatives were all healthy. Deletion of the paternal chromosome was the main genetic feature. Patients with paternal deficit in PWS are more common than those with maternal UPD. This result was in line with those of other studies (10, 11). Intrauterine hypotonia causes polyhydramnios, malpresentation, and reduced fetal movements, leading to a higher probability of cesarean. All these features were found in this study, in accord with other reports (12).

In the fetal period, PWS may manifest as fetal movement reduction, excessive amniotic fluid, and breech presentation. In the neonatal stage, marked hypotonia, weak cry, and feeding difficulties are characteristic clinical features (12). PWS is one of the leading causes of neonatal hypotonia (13). We collected the most accurate and detailed clinical information possible and found that all the seven cases showed poor sucking ability and central hypotonia. Therefore, if neonates present these two symptoms, especially in the presence of reduced intrauterine fetal movement, PWS should be considered. Beyhan Tuysuz and Gillessen-Kaesbach et al. found that PWS is commonly the cause of neonatal hypotonia, with a prevalence of 10.7–45% among infants with hypotonia (14, 15).

In this study, the results indicate that all seven cases were (nearly) full term and appropriate for GA, which was in accord with another Chinese study (11). It was reported that the full-term infants accounted for 80.4% of all 102 PWS patients. However, other studies have reported on the prevalence of preterm births and small for GA newborns (12). The discrepancies may be explained by the differences in sample size and ethnic differences. Specific genetic testing for PWS should be taken into account for all neonates with unknown central hypotonia, even in the absence of other major features of this syndrome. In this study, one PWS infant had a complication of mitochondrial myopathy, which had not been reported previously.

Patients with PWS often show a characteristic craniofacial morphology, including prominent forehead, narrow face, almond-shaped eyes, small mouth, and downturned mouth. However, craniofacial morphology are not obvious at birth. It become more typical with age (7). These symptoms are easy to miss if proper attention is not paid. Of the seven children in this study, we noted that five had one or more of these characteristic features. In addition, white skin and light hair are more conspicuous in patients of Asian origin. Small hands and feet and hypogonadism are also characteristic features of PWS (16). In fact, very few PWS patients are diagnosed in the neonatal period, and most of them are ignored and missed. The poor muscle tone gradually improves but persists in adulthood. Developmental delay, hyperphagia, rapid weight gain, and many behavioral problems gradually arise (17). PWS is commonly found after the onset of hyperphagia and obesity (18). It is important for clinicians to recognize the clinical continuum at different ages in the early diagnosis of this condition.

The relationship between PWS and PDA has not been well recognized until now. Abduljawad et al. (19) reported that PDA was the second most prevalent congenital heart disease, accounting for 25.5% of a total of 212 syndromic children. However, in their study, there was only one subject diagnosed with PWS. Olander et al. (20) reported a patient with maternal UPD and mosaic trisomy and 15 patients who had a more severe PWS phenotype with high morbidity of congenital heart disease. In this study, we found that three out of seven infants had PDA. Interestingly, there was one PDA infant of the two UPD patients, and the two infants with imprinting defect had PDA.

Infants with PWS are in a bad metabolic condition, often ignored in the early period. Harrington et al. reported that about 12.6% (12/95) of children with PWS develop hypoglycemia, possibly because of adrenal and GH deficiency (21). Ma et al. found five cases with hyperammonemia and two cases with hypoglycemia among nine PWS patients aged 10 days to 11 months (22). In this study, we found two cases of PWS with hypoglycemia and two with hyperammonemia. We speculated that the pathogenesis of hypoglycemia and hyperammonemia may be associated with endocrine metabolic abnormalities.

In this study, eyelid and limb edema were observed in one infant with PWS. Temporary eyelid and pedal edema were also previously reported in an infant case of PWS by Schmeling et al. (23). In fact, adult cases with PWS are often complicated with edema. The (U.S.) Prader-Willi Syndrome Association monitors revealed that edema is present in 16.78% of a total of 1,067 adult cases (24). The cause of the edema is mostly unclear but has frequently been attributed to obesity.

Since obesity-related complications are important causes of death in PWS cases, early diagnosis and careful follow-up are essential for the management of these patients. PWS has no cure so far. Prevention of obesity and appetite control are important therapeutic strategies. Early intervention, strict diet control, and GH therapy can improve quality of life (25). GH is a safe and effective drug to use for PWS treatment, as it promotes carbohydrate and fat metabolism, ensures the normal growth and development of children with PWS, controls body mass index, and improves cognitive function. It is often prescribed from 4 to 6 months old to adulthood (26). The annual mortality of PWS was about 3%, which is much higher compared with the general population. Respiratory failure caused by obesity has been reported to be the main cause of death in PWS patients (27). The treatment of PWS requires a multidisciplinary management involving rehabilitation, as well as endocrine, genetic, metabolic, psychological, and nutrition therapies. Different treatments have been adopted depending on the age group and endocrine disorders (28). These mainly include dietary behavior, nutrition management, management of cryptorchidism, sex hormone replacement therapy in adolescent patients, and use of GH (29). In our study, GH treatment started when the infant was 6 months old in order to increase the growth rate, reduce fat accumulation, improve muscle mass and strength, promote caloric consumption and protein synthesis, and increase bone density. GH is beneficial to improve the prognosis of PWS (30). Recently, it was found that topiramate may be helpful in the treatment of behavioral disorders. Topiramate had a significant effect on eating disorders, with a dose-effect relationship (31). Excitingly, CRISPR (clustered regularly interspaced short palindromic repeats) therapy and oligonucleotide therapy are now in the clinical validation phase of the American Food and Drug Administration (32).

The clinical features of PWS change with age. It is necessary for pediatricians to properly identify this disease. PWS is often misdiagnosed in neonates because of its nonspecific clinical manifestations. Pediatricians should recognize PWS in neonates with central hypotonia, feeding difficulty, and weak crying. It is advised to use a genetic method such as MS-MLPA in neonates with central hypotonia and feeding difficulties. Characteristic facial features and genital hypoplasia are also important features of PWS. Patent ductus arteriosus, hypoglycemia, hyperammonemia, and idiopathic edema may also be present in PWS patients. PWS can be promptly diagnosed in the neonatal period if proper attention is given to neonatal clinical features. Early diagnosis contributes to improve prognosis, and genetic testing is an effective diagnostic tool. The limitations of the study include the limited number of cases and the retrospective observational study design; therefore, further investigation are needed to confirm our results.

## Data Availability Statement

The original contributions presented in the study are included in the article/supplementary material, further inquiries can be directed to the corresponding author/s.

## Ethics Statement

The studies involving human participants were reviewed and approved by the Ethics Committee of the Shengjing Hospital of China Medical University (Protocol no. KYCS2019404). Written informed consent to participate in this study was provided by the participants' legal guardian/next of kin. Written informed consent was obtained from the individual(s), and minor(s)' legal guardian/next of kin, for the publication of any potentially identifiable images or data included in this article.

## Author Contributions

YH collected and analyzed the clinical data. XX searched literature. JF designed the study and was the major contributor in writing the manuscript. All authors contributed to the article and approved the submitted version.

## Conflict of Interest

The authors declare that the research was conducted in the absence of any commercial or financial relationships that could be construed as a potential conflict of interest.
